# Auditory Stream Segregation Can Be Modeled by Neural Competition in Cochlear Implant Listeners

**DOI:** 10.3389/fncom.2019.00042

**Published:** 2019-07-03

**Authors:** Andreu Paredes-Gallardo, Torsten Dau, Jeremy Marozeau

**Affiliations:** Hearing Systems Section, Department of Health Technology, Technical University of Denmark, Lyngby, Denmark

**Keywords:** auditory perception, auditory scene analysis, segregation, cochlear implant, computational neuroscience, auditory object perception, build-up

## Abstract

Auditory stream segregation is a perceptual process by which the human auditory system groups sounds from different sources into perceptually meaningful elements (e.g., a voice or a melody). The perceptual segregation of sounds is important, for example, for the understanding of speech in noisy scenarios, a particularly challenging task for listeners with a cochlear implant (CI). It has been suggested that some aspects of stream segregation may be explained by relatively basic neural mechanisms at a cortical level. During the past decades, a variety of models have been proposed to account for the data from stream segregation experiments in normal-hearing (NH) listeners. However, little attention has been given to corresponding findings in CI listeners. The present study investigated whether a neural model of sequential stream segregation, proposed to describe the behavioral effects observed in NH listeners, can account for behavioral data from CI listeners. The model operates on the stimulus features at the cortical level and includes a competition stage between the neuronal units encoding the different percepts. The competition arises from a combination of mutual inhibition, adaptation, and additive noise. The model was found to capture the main trends in the behavioral data from CI listeners, such as the larger probability of a segregated percept with increasing the feature difference between the sounds as well as the build-up effect. Importantly, this was achieved without any modification to the model's competition stage, suggesting that stream segregation could be mediated by a similar mechanism in both groups of listeners.

## Introduction

The cochlear implant (CI) is a neural prosthesis that allows many CI listeners to achieve high levels of speech understanding in quiet. Nevertheless, CI listeners typically experience difficulties to understand a single person's voice among many, or to recognize a familiar melody in a complex musical arrangement (e.g., Nelson et al., 2003). In such scenarios, the listener needs to parse the sounds in the complex auditory scene and group them into meaningful auditory objects or streams, a process known as auditory scene analysis (Bregman, 1990). However, the mechanisms that may allow CI listeners to perceptually group multiple sound events into streams remain unclear. The present study evaluated whether a computational model, proposed to account for the main aspects of auditory scene analysis observed in normal-hearing (NH) listeners, can also account for the behavioral data from CI listeners.

A common paradigm to investigate auditory scene analysis employs sequences of repeating, sequentially-presented sounds which may differ in various acoustic properties, typically the frequency content (for a review, see Carlyon, 2004; Micheyl and Oxenham, 2010; Gutschalk and Dykstra, 2014). Small differences and/or slow presentation rates promote the perceptual grouping of the sounds into a single stream (i.e., integration). Conversely, large differences and/or fast presentation rates promote the perceptual grouping of the sounds into several streams (i.e., segregation). The perception of the sequence has been described as bistable (e.g., Pressnitzer and Hupé, 2006) and it is characterized by ongoing spontaneous switches between an integrated and a segregated percept for long stimulus presentations. Nevertheless, the overall probability of experiencing a segregated percept has been reported to increase over time, typically reaching a plateau after the first couple of seconds. This phenomenon has often been referred to as the build-up of stream segregation (e.g., Bregman, 1978).

During the past decades, a variety of models have been proposed to account for the phenomenon reported in the experimental studies (see recent reviews by Szabó et al., 2016; Snyder and Elhilali, 2017). Based on a conceptual model described by Fishman et al. (2001), Rankin et al. (2015, 2017) proposed a neuromechanistic model to account for a variety of behavioral effects in NH listeners, including the effects of frequency differences and presentation rate, the dynamics of the bistable perception and the build-up effect. The model operates on the stimulus features at the cortical level and includes a competition stage between the neuronal units encoding the different percepts. The competition between the units results from a combination of mutual inhibition, adaptation and additive noise mechanisms, suggested to contribute to perceptual bistability at cortical stages (e.g., Moreno-Bote et al., 2007; Shpiro et al., 2009; Kondo et al., 2018).

Studies investigating the perceptual organization of sounds in CI listeners suggest that the listeners may be able to segregate sequential sounds on the basis of perceptual differences elicited by manipulations of the place, the rate or the intensity of the electrical stimulation (e.g., Cooper and Roberts, 2009; Marozeau et al., 2013; Paredes-Gallardo et al., 2018a,b,c). Furthermore, recent studies with CI listeners observed similar trends to those reported for NH listeners (albeit with a larger inter-subject variability), suggesting a common underlying mechanism in both groups of listeners (Paredes-Gallardo et al., 2018a,b,c).

The present study investigated whether neural competition at a cortical level, proposed to account for the behavioral effects of sequential stream segregation in NH listeners, can also account for the data from CI listeners. Specifically, the neuromechanistic model proposed by Rankin et al. (2015, 2017) was here used to account for the behavioral data from Paredes-Gallardo et al. (2018b,c). If the model would be able to capture the main trends in the behavioral data (i.e., the larger probability of a segregated percept with increasing the perceptual difference between the sounds and the build-up effect) without modifications to the competition stage, this would indicate that stream segregation can be described by a similar mechanism both in NH and CI listeners.

## Methods

### Behavioral Stream Segregation Data

Paredes-Gallardo et al. (2018b,c) investigated stream segregation in 7 and 9 CI listeners (respectively) making use of sequences of alternating A and B sounds. The sounds were encoded either via different electrodes at a constant pulse rate, or with different pulse rates from the same electrode, inducing in both cases a difference in perceived pitch (Δpitch). The listeners were asked to perform a temporal delay detection task that was easiest when the A and B sounds were perceptually segregated. Therefore, larger d' reflected a higher likelihood of a segregated percept. Overall, the d' scores increased with increasing the Δpitch between the A and B sounds, as well as with increasing the sequence duration. Thus, consistent with previous studies with NH listeners, the authors suggested that larger Δpitch might facilitate the perceptual segregation of the A and B sounds and that a segregated percept builds up over time.

### Stream Segregation Modeling Framework

**Figure 1:**
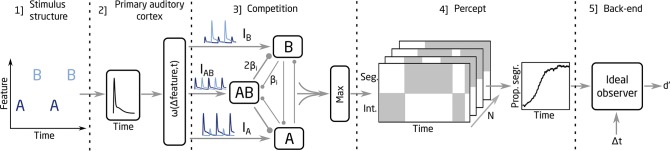
Schematic representation of the model.

The modeling framework used in the present study is based in the neuromechanistic model proposed by Rankin et al. (2015, 2017). A schematic representation of the framework is shown in Figure 1. The framework is divided into five parts: [1] The input to the model represents the dynamics of the stimulus (i.e., the onset times of the A and B sounds). [2] With this information, the model mimics the pulsatile responses and the feature dependence observed at the primary auditory cortex (Micheyl et al., 2005). A weighting function, ω*(*Δ*feature,t)*, is used in this stage to control the spread of the responses to three units in the competition network (represented by I_*A*_, I_*B*_ and I_*AB*_). [3] The competition between the units is modeled through a combination of mutual inhibition, recurrent excitation, slow adaptation and noise, which is added to I_*A*_, I_*B*_, and I_*AB*_. The inhibition processes, proportional to the inhibition strength parameter β_*i*_, are indicated with round-ended connectors in Figure 1. The recurrent excitation, slow adaptation, and additive noise are not shown in Figure 1. The model encodes integration when the activity of the AB unit is larger than the activity from the A and the B units and a segregated percept otherwise. Thus, the output from the competition network is a binary representation of the percept over time. [4] The build-up function is then computed by time-binned averaging across N simulations, which represents the time course of the proportion of segregation over N trials. [5] To link the proportion of segregation with the d' scores, an ideal observer (IO) was used in the back-end of the model. The IO assumed a 100% hit rate for the segregated trials and chance level performance for the integrated trials, estimating a d' score for a given sequence duration, Δpitch, and Δt (for more details on the IO model, see Paredes-Gallardo et al., 2018b,c).

### Model Parameters and Fitting Procedure

Rankin et al. (2017) proposed the neuromechanistic model to account for behavioral data from NH listeners. The acoustic stimuli consisted of repeating triplets of ABA sounds separated by a short pause (ABA_). The model parameters were defined to minimize the deviations between the model predictions and the behavioral data. In the present study, unless otherwise specified, the model equations and parameters were kept as described in Rankin et al. (2017). However, the stimulus structure was slightly modified to resemble the stimuli used by Paredes-Gallardo et al. (2018b,c). In addition, some parameters in the second part of the model, related to the input signals to the competition stage, were adjusted to account for the differences between the input signals in NH and CI listeners.

In the original model, the A and B sounds were defined as pure tones with different frequencies. Thus, the weighting function ω(Δ*feature, t*) was dependent on the frequency difference between the sounds [i.e., ω(Δ*f,t)*]. Conversely, in the studies from Paredes-Gallardo et al. (2018b,c), the A and the B sounds differed either in the place or the rate of the electrical stimulation, eliciting a difference in the perceived pitch. Thus, in the present study, the dependency of the weighting function on the frequency difference was replaced by a dependency on Δpitch, as indicated by Equation (1). The variable *t* represents the time vector, *L* the amplitude factor and σ the lateral decay constant. Q(t) and R(t) are exponential decay functions and represent the amplitude and the Δpitch adaptation of the input, respectively, with a time constant of 500 ms (for more details, see Rankin et al., 2017).

(1) $$\begin{array}{r} \omega \left(\Delta pitch,t\right)=\;Q\left(t\right)Le^{\left(\frac{-R\left(t\right)pitch}{\sigma }\right)} \end{array}$$

In the study from Rankin et al. (2017), the lateral decay constant σ was defined in semitones. As a result of the change in the dependency of ω from frequency separation to Δpitch, σ had to be redefined in the present study. Two different model fits were considered here. For the first one, a genetic algorithm was used to find the value of σ leading to the minimum averaged mean error (AME) between the mean d' scores achieved by the listeners and the model predictions. For the second one, the genetic algorithm was allowed to adjust the value of the amplitude factor *L* in addition to σ in order to minimize the AME between the predictions and the data. In both cases, the fitting of the model was performed by manipulating the parameters of the weighting function, and no changes were made to the parameters or equations from the competition stage. The values of σ and the AME resulting from the fitting procedure are presented rounded to the first significant figure. The value of L is presented rounded to the second significant figure.

The simulations were performed on a sequence of alternating A and B sounds with a presentation rate of 5.89 Hz. Each d' estimate was computed from 1,000 simulated trials (N) at 1.24 and 3.96 s (i.e., equivalent to the long and the short sequence durations from Paredes-Gallardo et al.). Δt was set to 48.5 ms, the median value across the listeners from Paredes-Gallardo et al. (2018b,c).

## Results

Two different model fits were considered in the present study: one where only σ was adjusted and L was fixed to 0.6, as in Rankin et al. (2017), and another one where both σ and *L* were adjusted. When only σ was adjusted, the minimum AME between the predictions and the data was achieved for σ = 40 (AME = 0.5). Conversely, when both σ and *L* were adjusted, the minimum AME was achieved for σ = 30 and L = 0.35 (AME = 0.3).

**Figure 2:**
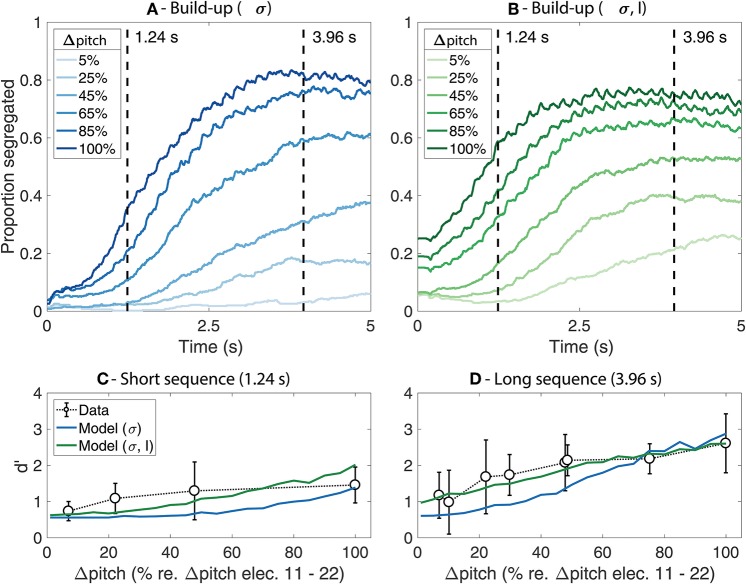
**(A,B)** Simulated build-up functions computed by time-binned averaging across trials. Lighter colors represent smaller Δpitch values whereas darker colors represent larger Δpitch values. The simulations from the model where only σ was adjusted are presented in blue **(A)** whereas the simulations from the model where both σ and *L* were adjusted are presented in green **(B)**. The duration of the stimuli is indicated with dashed vertical lines. **(C,D)** Comparison between the mean d' from the behavioral data (open markers) and the model predictions (solid lines) for the short **(C)** and the long **(D)** stimuli. The error bars represent ±1 standard deviation.

Figure 2 shows the predicted proportion of segregation as a function of time (i.e., the build-up functions) for each of the model fits (Figures 2A,B) as well as a comparison between the predicted d' values and the behavioral data for the long and the short sequence durations (Figures 2C,D). The results from the simulations where only the value of σ was adjusted are shown in blue whereas the results from the simulations where both σ and *L* were adjusted are shown in green.

In Figures 2A,B, lighter colors indicate smaller Δpitch and darker colors indicate larger Δpitch. As an arbitrary reference, Δpitch of 100% represents the perceptual pitch difference between a 900 pps pulse train stimulating electrode 11 vs. electrode 22 of the array. Similar trends are observed for both model fits. The proportion of segregation increases over time and reaches a plateau after ~2–~4 s for most Δpitch conditions. The plateau of the build-up functions happens at values below 1, suggesting the presence of perceptual switches between an integrated and a segregated percept throughout the trial. Larger Δpitch values lead to steeper slopes, reaching the plateau in a shorter time (i.e., faster build-up). Nevertheless, the effect of both Δpitch and time is more pronounced in Figure 2A than in Figure 2B, where the amplitude factor *L* was set to 0.35, a smaller value than the original value of 0.6 in the model for NH listeners.

The comparison between the predicted d' scores from the model and the d' scores from the listeners are shown in Figure 2C (short sequence) and Figure 2D (long sequence). The solid lines represent the model predictions, and the open markers represent the mean d' scores from the listeners. The error bars indicate ±1 standard deviation. The d' scores achieved by the listeners generally increase with increasing Δpitch between the sounds and are, overall, higher for the long than for the short sequences, reflecting the build-up effect. In addition, the effect of Δpitch was larger for the long than for the short sequence. These trends are well-captured by the model, both for the fit where only σ was adjusted and for the fit where σ and *L* were manipulated. Nevertheless, a better agreement between the data and the predictions is observed when adjusting the value of the amplitude factor *L* in addition to σ (solid green line). However, whereas the d' scores achieved by the listeners saturate for large Δpitch values both for the long and the short sequences, the predictions from the model continue to increase at large Δpitch values for the short sequence. This can also be seen in the build-up functions from Figures 2A,B: the proportion of segregation saturates with increasing Δpitch in the plateau region of the build-up functions (i.e., for the long sequence) and continues to increase with increasing Δpitch in the steep region of the build-up functions (i.e., for the short sequence).

## Discussion

The present study evaluated whether the neuromechanistic model proposed by Rankin et al. (2015, 2017) would be able to account for the effects observed in the behavioral data from CI listeners. The model parameters and equations from the competition stage were kept as defined in Rankin et al. (2017), and only the function defining the amount of input spread to the different units from the competition network, ω(Δ*feature,t)*, was adjusted to account for the data from the CI listeners. Specifically, the model parameters σ and *L* were adjusted. The adjustment of σ was a necessary step in order to change the dependency of ω from frequency separation to Δpitch. When σ was adjusted, and the remaining model parameters were kept as defined by Rankin et al. (2017) for NH listeners, the model was able to capture the main trends of the behavioral data from the CI listeners (i.e., larger d' scores with increasing Δpitch and the sequence duration). Nevertheless, a better fit between the data and the model predictions was achieved when modifying the amplitude factor *L* in addition to σ. The optimal value for *L* was found to be 0.35, a lower value than the value of 0.6 used by Rankin et al. (2017). The lower input amplitude *L* reduces the effect of Δpitch on the proportion of segregation and increases the relative weight of the additive noise in the model, resulting in a more ambiguous percept (i.e., higher minima and lower maxima of the build-up functions in Figure 2B with respect to those from Figure 2A). Such ambiguity could arise from the generally weak pitch percept experienced by CI listeners (e.g., Oxenham, 2008). Thus, the findings from the present study suggest that a more ambiguous percept may better characterize the behavioral data from CI listeners, which may indicate a weaker role of obligatory processes on stream segregation in CI listeners than in NH listeners.

Even though the model successfully captures the main trends of the data, there are some discrepancies between the data and the model predictions. Specifically, whereas the d' scores achieved by the listeners saturate for large Δpitch values both for the long and the short sequences, the model only predicts a saturation effect for the long sequence (i.e., in the plateau region of the build-up function). The agreement between the model predictions and the behavioral data could be further improved by manipulating model parameters affecting the build-up process [e.g., by adjusting or redefining the exponential decays R(t) and Q(t) from Equation (1)]. However, a better behavioral characterization of the build-up functions for CI listeners would be required to support such modifications in the model. The aim of the present study was not to achieve the best fit between the model predictions and the data but to evaluate whether a model that was proposed to account for data from NH listeners can capture the main trends in the data from CI listeners. Overall, the model was able to account for the effect of perceptual pitch differences (Δpitch) elicited by changes in the electrode or the pulse rate of the electrical stimulation on stream segregation, as well as the build-up effect. Importantly, this was achieved without any changes to the model parameters and without modifying the characteristics of the competition stage. These findings indicate that a competition network featuring mutual inhibition, adaptation and additive noise can account for the behavioral effects of stream segregation, also in CI listeners, suggesting that stream segregation may be mediated by a similar mechanism in NH and CI listeners.

Finally, the results presented in this study are consistent with findings from invasive physiological studies in animals and modeling work suggesting that many important aspects of stream segregation, such as the effect of perceptual differences between the sounds or the build-up effect may be explained by relatively basic neural mechanisms at a cortical level (e.g., Fishman et al., 2001, 2017; Micheyl et al., 2005, 2007). Nevertheless, more experimental data from CI listeners are needed to evaluate whether the neuromechanistic model can account for a wider range of behavioral effects of stream segregation in CI listeners, such as the effects of variations in the stimulus presentation rate or the dynamics of bistable perception.

## Data Availability

The simulations generated for this study can be found at http://doi.org/10.5281/zenodo.2577966. The data from Paredes-Gallardo et al. (2018b,c) is publicly available at http://doi.org/10.5281/zenodo.1211629 and http://doi.org/10.5281/zenodo.890791.

## Author Contributions

AP-G designed and performed the research. AP-G, TD, and JM interpreted the results and wrote the manuscript.

### Conflict of Interest Statement

The authors declare that the research was conducted in the absence of any commercial or financial relationships that could be construed as a potential conflict of interest.

## References

[B1] Bregman A. S. (1978). Auditory streaming is cumulative. J. Exp. Psychol. Hum. Percept. Perform. 4, 380–387. 10.1037/0096-1523.4.3.380 681887

[B2] Bregman A. S. (1990). Auditory Scene Analysis: The Perceptual Organization of Sound. Cambridge, MA: The MIT Press.

[B3] Carlyon R. P. (2004). How the brain separates sounds. Trends Cogn. Sci. 8, 465–471. 10.1016/j.tics.2004.08.008 15450511

[B4] Cooper H. R. Roberts B. (2009). Auditory stream segregation in cochlear implant listeners: measures based on temporal discrimination and interleaved melody recognition. J. Acoust. Soc. Am. 126, 1975–1987. 10.1121/1.3203210 19813809

[B5] Fishman Y. I. Kim M. Steinschneider M. (2017). A crucial test of the population separation model of auditory stream segregation in macaque primary auditory cortex. J. Neurosci. 37, 10645–10655. 10.1523/JNEUROSCI.0792-17.2017 28954867 PMC5666585

[B6] Fishman Y. I. Reser D. H. Arezzo J. C. Steinschneider M. (2001). Neural correlates of auditory stream segregation in primary auditory cortex of the awake monkey. Hear. Res. 151, 167–187. 10.1016/S0378-5955(00)00224-0 11124464

[B7] Gutschalk A. Dykstra A. R. (2014). Functional imaging of auditory scene analysis. Hear. Res. 307, 98–110. 10.1016/j.heares.2013.08.003 23968821

[B8] Kondo H. M. Pressnitzer D. Shimada Y. Kochiyama T. Kashino M. (2018). Inhibition-excitation balance in the parietal cortex modulates volitional control for auditory and visual multistability. Sci. Rep. 8, 1–13. 10.1038/s41598-018-32892-3 30267021 PMC6162284

[B9] Marozeau J. Innes-Brown H. Blamey P. (2013). The effect of timbre and loudness on melody segregation. Music Percept. 30, 259–274. 10.1525/mp.2012.30.3.259

[B10] Micheyl C. Carlyon R. P. Gutschalk A. Melcher J. R. Oxenham A. J. Rauschecker J. P.. (2007). The role of auditory cortex in the formation of auditory streams. Hear. Res. 229, 116–131. 10.1016/j.heares.2007.01.007 17307315 PMC2040076

[B11] Micheyl C. Oxenham A. J. (2010). Pitch, harmonicity and concurrent sound segregation: psychoacoustical and neurophysiological findings. Hear. Res. 266, 36–51. 10.1016/j.heares.2009.09.012 19788920 PMC2885481

[B12] Micheyl C. Tian B. Carlyon R. P. Rauschecker J. P. (2005). Perceptual organization of tone sequences in the auditory cortex of awake macaques. Neuron 48, 139–148. 10.1016/j.neuron.2005.08.039 16202714

[B13] Moreno-Bote R. Rinzel J. Rubin N. (2007). Noise-induced alternations in an attractor network model of perceptual bistability. J. Neurophysiol. 98, 1125–1139. 10.1152/jn.00116.2007 17615138 PMC2702529

[B14] Nelson P. B. Jin S.-H. Carney A. E. Nelson D. A. (2003). Understanding speech in modulated interference: cochlear implant users and normal-hearing listeners. J. Acoust. Soc. Am. 113, 961–968. 10.1121/1.1531983 12597189

[B15] Oxenham A. J. (2008). Pitch perception and auditory stream segregation: implications for hearing loss and cochlear implants. Trends Amplif. 12, 316–331. 10.1177/1084713808325881 18974203 PMC2901529

[B16] Paredes-Gallardo A. Innes-Brown H. Madsen S. M. K. Dau T. Marozeau J. (2018a). Auditory stream segregation and selective attention for cochlear implant listeners: evidence from behavioral measures and event-related potentials. Front. Neurosci. 12:581. 10.3389/fnins.2018.00581 30186105 PMC6110823

[B17] Paredes-Gallardo A. Madsen S. M. K. Dau T. Marozeau J. (2018b). The role of temporal cues on voluntary stream segregation in cochlear implant users. Trends Hear. 22:2331216518773226. 10.1177/2331216518773226 29766759 PMC5974563

[B18] Paredes-Gallardo A. Madsen S. M. K. Dau T. Marozeau J. (2018c). The role of place cues in voluntary stream segregation for cochlear implant users. Trends Hear. 22:233121651775026. 10.1177/2331216517750262 29347886 PMC5777547

[B19] Pressnitzer D. Hupé J.-M. (2006). Temporal dynamics of auditory and visual bistability reveal common principles of perceptual organization. Curr. Biol. 16, 1351–1357. 10.1016/j.cub.2006.05.054 16824924

[B20] Rankin J. Osborn Popp P. J. Rinzel J. (2017). Stimulus pauses and perturbations differentially delay or promote the segregation of auditory objects: psychoacoustics and modeling. Front. Neurosci. 11:198. 10.3389/fnins.2017.00198 28473747 PMC5397483

[B21] Rankin J. Sussman E. Rinzel J. (2015). Neuromechanistic model of auditory bistability. PLoS Comput. Biol. 11:e1004555. 10.1371/journal.pcbi.1004555 26562507 PMC4642990

[B22] Shpiro A. Moreno-Bote R. Rubin N. Rinzel J. (2009). Balance between noise and adaptation in competition models of perceptual bistability. J. Comput. Neurosci. 27, 37–54. 10.1007/s10827-008-0125-3 19125318 PMC2913428

[B23] Snyder J. S. Elhilali M. (2017). Recent advances in exploring the neural underpinnings of auditory scene perception. Ann. N. Y. Acad. Sci. 1396, 39–55. 10.1111/nyas.13317 28199022 PMC5446279

[B24] Szabó B. T. Denham S. L. Winkler I. (2016). Computational models of auditory scene analysis: a review. Front. Neurosci. 10:524. 10.3389/fnins.2016.00524 27895552 PMC5108797

